# Psychosocial risks emerged from COVID-19 pandemic and workers’ mental health

**DOI:** 10.3389/fpsyg.2023.1148634

**Published:** 2023-05-26

**Authors:** Helena Koren, Marina Milaković, Marija Bubaš, Petra Bekavac, Barbara Bekavac, Lovro Bucić, Jelena Čvrljak, Magdalena Capak, Pavle Jeličić

**Affiliations:** ^1^Division for Occupational Health, Croatian Institute of Public Health, Zagreb, Croatia; ^2^Division for Environmental Health, Croatian Institute of Public Health, Zagreb, Croatia; ^3^Division for Public Health, Andrija Stampar Teaching Institute of Public Health, Zagreb, Croatia; ^4^Department for History, Croatian Catholic University, Zagreb, Croatia

**Keywords:** psychosocial risks, workplace stress, mental health, COVID-19, pandemic

## Abstract

This paper examines the impact of work in a pandemic context on workers’ mental health. Psychosocial risks have always been a challenging aspect of workplace health and safety practices. Moreover, the COVID-19 pandemic has affected workplaces in all sectors causing unexpected changes in work organization and working conditions, leading to the emergence of new psychosocial risks for health and wellbeing of workers. This mini-review aims to identify the main work stressors during pandemic period and related mental health problems to suggest recommendations and adjust health and safety practices regarding workplace mental health. A literature search has been performed using MEDLINE/PubMed, ResearchGate and Google Scholar databases, selecting articles focusing on work-related stressors and workers’ mental health problems related to the pandemic. Specific psychosocial risks have been identified, including fear of contagion, telework-related risks, isolation and stigmatization, rapid digitalization demands, job insecurity, elevated risk of violence at work or home, and work-life imbalance, among others. All those risks can lead to elevated levels of stress among workers and affect their mental health and wellbeing, especially in terms of psychological distress, anxiety, and depression. As one of the social determinants of health, the workplace has an important and moderating role in workers’ health. Therefore, in the pandemic context more than ever health protection practices at the workplace should be devoted to mental health problems. Recommendations provided in this study are expected to contribute to workplace practices to preserve and promote workers’ mental health.

## Introduction

Since the World Health Organization (WHO) in 2020 declared the coronavirus outbreak an international public health emergency, which was soon declared as a pandemic (World Health Organization, 2020a) peoples’ lives have changed in many ways. Fear and uncertainty, adaptation to new ways of living and working and consequently high levels of perceived stress have affected peoples’ mental health. In the context of work, COVID-19 pandemic has affected workplaces across different sectors causing unexpected changes in work organization and working conditions, leading to the emergence of new psychosocial risks to workers’ health and wellbeing (International Labour Organization, 2020a). Psychosocial risks have always been a complicated aspect of workplace health and safety practices (European Agency for Safety and Health at Work, 2012). However, the pandemic has exposed workers to diverse psychosocial hazards impacting their health and wellbeing. Therefore, the aim of this mini-review is to identify the main work stressors during the pandemic period and related psychological and mental health problems among the most affected working groups, such as healthcare professionals and teleworkers.

Psychosocial hazards are defined as “those aspects of work design, work organization and management, and their social and environmental context that could potentially cause physical or psychological harm” (Cox et al., 2000). According to Cox et al. (2000), the sources of psychosocial risks are numerous: job content, workload and work pace working hours and shift work, workers’ autonomy, control and participation in decision-making, organization climate including poor communication, poor leadership and perceived injustice, job insecurity, role problems such as ambiguity or role conflicts, interpersonal relationships, lack of social support or adverse social behaviors, such as harassment and violence, work-life imbalance, among others.

Psychosocial risks can affect workers’ physical and psychological wellbeing through stress experience.

According to WHO, work-related stress is “the response people may have when presented with job demands and pressures that do not match their knowledge or skills and which challenge their coping abilities” (World Health Organization, 2020b). During the pandemic, workers’ regular abilities to cope may have been exceeded in front of new pandemic-related psychosocial hazards, which could have resulted in high levels of work-related stress. Especially situations such as emergencies can lead to a state of chronic stress in which person may feel overwhelmed or unable to cope (World Health Organization and the International Labour Organization, 2018).

Many occupational groups have been directly affected by the pandemic. To large extent frontline workers responding to health emergencies could have experienced many sources of stress, such as the lack of personal protective equipment (PPE), consequences of wearing PPE, the fear of being infected and infecting family members, the conflict between safety procedures and providing care or performing tasks, long working hours, multitasking and the stigmatization of those working in high-risk environments (World Health Organization and the International Labour Organization, 2018; Giorgi et al., 2020). Workers employed in activities necessary for functioning during the pandemic (e.g., healthcare workers, police and civil protection, or services like delivery, transport or shops) have been exposed to numerous stressful situations. On the other side, many workers suddenly had to switch to telework and work in a home-setting, which has exposed them to different psychosocial risks such as balancing responsibilities of work, childcare and homeschooling, unstructured working time, imbalance between private and work part of the day and rapid digitalization (Bouziri et al., 2020; International Labour Organization, 2020a). It is important to note that one part of the workers faced an exceptional stressor such as the fear of losing their job or a circumstance of a job loss due to business closure during lockdown (International Labour Organization, 2020a).

Undoubtedly, work during the pandemic has been perceived as uncertain and stressful, causing a wide range of stress responses in workers, and consequently mental problems like mood changes, exhaustion, anxiety and depression, burnout and suicidal thoughts as well as reduced motivation and behaviors such as increased use of alcohol, tobacco, and other unhealthy habits (Stansfeld and Candy, 2006; International Labour Organization, 2020a). Regarding mental health, the COVID-19 pandemic is likely to exacerbate existing symptoms or worsen pre-existing mental health problems (International Labour Organization, 2020a). People with mental health problems could have difficulties to cope with multiple stressors related to the pandemic. According to the newest WHO statistics it is suggested that the pandemic has triggered an increase of 25% in the prevalence of anxiety and depression (World Health Organization, 2022). Data suggest that women tend to report higher levels of anxiety and depression in normal times and in emergencies. Possible explanation could be over-representation of women in more affected sectors (such as services) and frontline occupations (such as healthcare workers, e.g., nurses). Furthermore, women experience more burden of childcare and care for other members of a family, as well as household tasks (International Labour Organization, 2020a; International Labour Organization, 2020b). On the other side, men, especially if they are expected to provide family finances, have vulnerabilities related to job insecurity and loss of employment.

Identifying and assessing new psychosocial risks that emerged from pandemics, related mental health problems and groups at risk is the key step for implementing preventive measures to protect the health and wellbeing of workers in the context of the COVID-19 pandemic.

## Methodology

A literature search was performed between September and November 2022 using MEDLINE/PubMed and ResearchGate databases as well as Google Scholar search engine. The search was restricted to recent articles, published since January 2020 and in English language, focusing on psychosocial risks, workplace stress, and workers’ mental health problems, all in relation to the pandemic context. Initial search was performed using terms “psychosocial risks AND pandemic,” “workplace stress AND pandemic,” “work AND pandemic” and “mental health AND pandemic.” In order to obtain more articles, the term “pandemic” was substituted with “COVID-19.” Furthermore, a manual search of references to extend the search was performed. Finally, available full-text articles focusing on work-related psychosocial risks and related mental health in the context of the COVID-19 pandemic were considered for this mini-review. Besides scientific articles, relevant publications containing guidelines, recommendations or interventions for the work population published by recognized institutions such as WHO or International Labor Organization (ILO) were included, as well as articles explaining broader context of the research topic.

## Psychosocial risks emerged from the pandemic

Although publications presented numerous psychosocial factors arising from the pandemic, for purpose of this article specific work-related psychosocial hazards will be discussed, including fear of contagion, stigmatization, telework-related risks, isolation, rapid digitalization demands, job insecurity, elevated risk of violence at work or home and work-life imbalance.

During the COVID-19 pandemic, many workers have been exposed to a greater likelihood of being infected. Fear of infection, as well as workers’ perception that their health and safety was threatened by their work environment could have generated work-related stress through workers’ awareness, suspicion or fear that they were exposed to harm (Levi, 1984; Cox et al., 2000). In the pandemic context, exposure to the potentially dangerous virus, in a combination with a lack of information and, in some cases, lack of protective measures and equipment, could have caused stress among workers. Fear of contagion was the most common among frontline workers, such as healthcare and medical workers, workers in jobs that require contact with the public, workers in shops, restaurants, public services, school or transport service, as well as workers in sectors that had to continue to work in high-density environments such as factories or call centers (International Labour Organization, 2020a).

Related to the fear of contagion, a highly present psychosocial hazard during the pandemic was stigmatization. Social stigma in the context of the pandemic is “the negative association between a person or a group of people who share certain characteristics and a specific disease” (World Health Organization, 2022). WHO stated that during an outbreak stigma may cause people to be labeled, discriminated against and treated separately because of a perceived association with a disease (World Health Organization, 2020c). International Labour Organization (2020a) confirmed that work-related violence and harassment tend to rise during infectious disease outbreaks. Discriminatory behaviors related to increased social stigma could have contributed to work-related violence and harassment, both physical and psychological, which is considered to be psychosocial risk of high importance. In addition, the literature suggested that the most exposed to discrimination and stigma were infected people and healthcare workers (Giorgi et al., 2020).

On the other hand, many workers during the pandemic had to change their usual workplaces for home settings, exposing them to different telework-related risks. Inadequate ergonomic or working conditions (such as noise from the household) and factors like inadequate work equipment could have resulted in increased levels of stress. Further psychosocial risks associated to telework, such as unstructured working time, isolation and blurred boundaries between private and work part of the day, limited social interactions, domestic tasks and childcare created extra stress and difficulties in balancing work and family responsibilities (Bouziri et al., 2020; International Labour Organization, 2020a). Kotera and Vione (2020) suggested that new ways of work, including telework, could have impacted positively workers’ work engagement, work-related flow, and connections between employees. However, telework could have also negatively impacted workers’ psychological state increasing mental demands and causing fatigue. A study among Brazilian workers found differences between workers who voluntarily teleworked before the COVID-19 pandemic and those who did not have telework experience before the pandemic (El Kadri Filho and de Lucca, 2022). Workers with previous telework experience declared reduced ergonomic and psychosocial risks (El Kadri Filho and de Lucca, 2022). This led to conclusion that obligatory and unprepared switch to telework could have been especially stressful for workers.

Furthermore, digitalization of work is part of normal and expected technological change, however, that change has never been more rapid than during the pandemic. The pandemic required rapid adaptation to nonstandard ways of performing job tasks. Although it was shown that digitalization reduced costs and increased efficiency and information sharing among colleagues (El Kadri Filho and de Lucca, 2022), digitalization also triggered an intensification of work, increasing time pressures and disrupting social contact among workers (Palumbo and Cavallone, 2022). Digitalization and the rapid need for new skills development became a psychosocial hazard, especially for those workers whose skills did not match job demands. In addition, it was noticed that working from home also increased domestic violence. Studies suggested that forced proximity, along with economic stress and emergency related instability, were risk factors for aggression and home violence (Pedrosa et al., 2020).

Finally, changes in workload have affected workers in different ways. Frontline workers were exposed to augmented workload leading to symptoms of anxiety, depression and burnout. However, a certain part of workers had reduced workloads during the pandemic which was related to loss of economic status and consequently poor mental outcomes. As well, pandemic-related uncertainty manifested in job insecurity and economic problems, which was a major work-related stressor. Authors declared that uncertainty about the future and the lack of guaranteed employment were associated with increased stress, anxiety, depression and burnout (Kim and von dem Knesebeck, 2015). Finally, remote workers could have experienced technostress and home–work imbalance.

Similarly, Lulli et al. (2021) identified five important topics related to psychosocial aspects in the workplace during the COVID-19 pandemic: job insecurity and financial stress, work competence and adequate training (especially in healthcare workers), changes in workload and job demand, home-work balance and finally, support from colleagues and organization being a protective factor for mental health.

Ultimately, regarding potential work-related stressors in the post-pandemic period, predictions can be made based on previous pandemic outcomes. Authors pointed out that besides posttraumatic stress disorder related to the recovery from a life-threatening illness, it seemed that identified factors such as stigmatization, financial issues and job insecurity may have a long-lasting effect after COVID-19 (Hamouche, 2020).

## Mental health during the pandemic and groups at risk

As mentioned before, working in the pandemic was characterized by high uncertainty, fear and high levels of stress. Thus, different authors reported a range of mental health problems among workers. The main reported mental health problems related to the pandemic were stress, anxiety, depression, insomnia, denial, anger, fear, post-traumatic stress disorder and sleep disorders, alcohol, and drug misuse (Giorgi et al., 2020; Gaspar et al., 2021). It was also shown that mental issues related to the pandemic were more likely to affect healthcare and emergency workers, migrant workers, young workers, workers in contact with the public and people with existing mental illnesses (Giorgi et al., 2020; Gaspar et al., 2021).

The majority of the selected studies on occupational stress during the pandemic time considered healthcare workers (Giménez-Espert et al., 2020; Huang and Zhao, 2020; Morgantini et al., 2020; Ruiz-Fernández et al., 2020; Stelnicki et al., 2020; Wang et al., 2020; Franklin and Gkiouleka, 2021; Galbraith et al., 2021; Herraiz-Recuenco et al., 2022; Moreno Martínez et al., 2022; Tomaszewska et al., 2022), showing that they are a high-risk group for developing mental health problems derived from the pandemic (Giorgi et al., 2020; Franklin and Gkiouleka, 2021). For example Gaspar et al. (2021) found that health professionals were among those who suffer most from psychological stress and had the highest risk of burnout, and consequently greater risk of long-term symptoms, specifically chronic stress, depression and anxiety, increased substance use and finally, absenteeism from work. Furthermore, Franklin and Gkiouleka (2021) identified, except anxiety and depression disorders, symptoms of psychological trauma and posttraumatic stress, sleep disturbances, insomnia and fatigue, psychical and emotional exhaustion and burnout among healthcare workers. More specific data among the Spanish healthcare workforce showed high percentages of healthcare workers suffering from major depressive disorders (28.1%), generalized anxiety disorders (22.5%), panic attacks (24.0%), post-traumatic stress disorders (22.2%), and substance use disorders (6.2%) (Alonso et al., 2021). Similarly, research on healthcare workers in Italy showed that 49.38% of them had post-traumatic stress symptoms, 24.73% had symptoms of depression, 19.80% symptoms of anxiety, 8.27% insomnia and 21.90% high perceived stress (Rossi et al., 2020). Those are worrisome data indicating an urgent need to offer support for most exposed frontline workers during the pandemic.

On the other side, researches on working from home showed both positive and negative effects of telework on mental health and quality of life (Lulli et al., 2021; Platts et al., 2022; Zhang and Chen, 2022). However, one of the most important factors to be considered is that in the pandemic context, telework was enforced for a large number of workers, and the effect of mandatory telework could be much different than voluntary or optional, especially in the context of lockdown, restrictions and limited social interactions. One of the studies of mandatory telework during the pandemic (Platts et al., 2022) showed that telework could have had more negative impact on workers with existing mental health conditions. In those without mental health conditions, more stress and depressive symptoms were experienced by women and workers under 45 years (Platts et al., 2022). Women in general seemed to be more affected by the pandemic and telework than men.

**Figure 1:**
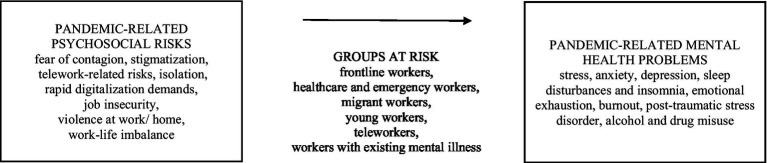
Main pandemic-related psychosocial risks and mental health problems.

The main pandemic-related psychosocial risks and mental health problems are summarized in Figure 1.

## Workplace as determinant of health

Work is considered to be social determinant of health (Wilkinson and Marmot, 2003), one of the non-medical factors that influence health outcomes in positive and negative way (World Health Organization, 2023). It can expose workers to different hazards from work environment, however, it can be beneficial for workers’ health assuring healthy physical and psychosocial working conditions. Therefore, occupational health and safety practitioners should consider the social context of work in order to minimize the negative effects of work to workers’ health and foster the positive ones. Furthermore, in the pandemic context more than ever health protection practices at the workplace should be devoted to mental health problems.

World Health Organization and International Labour Organization (2022) recommends an integrative approach to the management of mental health at the workplace, focusing on three main aspects: (a) prevention, (b) protection and promotion, and (c) support. Key interventions regarding prevention consider adequate psychosocial risk assessment intending to minimize those risks and prevent workers from experiencing work-related stress and mental health problems. The aspect of protection and promotion includes raising awareness and strengthening skills, recognizing and early acting on mental health issues to protect and promote the mental health of all workers, mostly through education and training. It could also include activities toward enhancing employees’ resilience and better stress-coping strategies. Finally, support considers activities toward workers with mental health problems to continue working. In general, activities addressing different aspects of the work environment (organizational measures) combined with individual interventions are shown to be the most effective solution to prevent psychosocial risks at work (Eurofound and EU-OSHA, 2014).

Referring to specific measures, it was found that during the COVID pandemic, when occupational stress was at very high levels, peer support was a key factor for managing work related stress. Another important factor related to workers’ mental wellbeing was organizational support, which refers to employees’ global beliefs regarding “the extent to which the organization values their contributions and cares about their wellbeing” (Kim and von dem Knesebeck, 2015). Social support at work was recognized throughout the literature as a protective factor against occupational stress, mitigating negative effects of high job strain, therefore it was beneficial for mental health (Karasek and Theorell, 1990; Hamouche, 2020). Furthermore, measures that were found to be successful in managing psychological distress among healthcare workers during past outbreaks included clear communication, access to adequate personal protection, adequate rest, and both practical and psychological support (Kisely et al., 2020).

## Conclusion

From the perspective of occupational health and safety, although the pandemic has exposed workers to new risks and increased levels of stress, it has also raised awareness about the need to manage work-related stress and mental health problems. Creating healthy workplaces and a positive psychosocial environment is the way that employers can foster workers’ resilience and promote mental health, especially in times of emergencies. This article provides employers, occupational health and safety specialists and stakeholders with key factors to consider in psychosocial risk assessment in the pandemic context in order to implement measures for protection and promotion of workplace mental health based on so far known information. However, not all long-term psychosocial consequences can be known at this moment, so further research will be needed as the situation evolves.

## Author contributions

HK, MM, and MB contributed to conception and design of the study. MC, JČ, LB, and BB performed databases search. BB and PB selected and organized the relevant articles. HK and MM wrote the first draft of manuscript. HK, MM, MB, PB, and PJ wrote sections of the manuscript. All authors reviewed the manuscript and approved the final version.

## Conflict of interest

The authors declare that the research was conducted in the absence of any commercial or financial relationships that could be construed as a potential conflict of interest.

## Publisher’s note

All claims expressed in this article are solely those of the authors and do not necessarily represent those of their affiliated organizations, or those of the publisher, the editors and the reviewers. Any product that may be evaluated in this article, or claim that may be made by its manufacturer, is not guaranteed or endorsed by the publisher.
